# Population structure analysis of *Phlebotomus papatasi* populations using transcriptome microsatellites: possible implications for leishmaniasis control and vaccine development

**DOI:** 10.1186/s13071-024-06495-z

**Published:** 2024-10-02

**Authors:** Omar Hamarsheh, Souad Guernaoui, Mehmet Karakus, Mohammad Reza Yaghoobi-Ershadi, Andreas Kruger, Ahmad Amro, Mohamed Amin Kenawy, Mostafa Ramadhan Dokhan, Douglas A. Shoue, Mary Ann McDowell

**Affiliations:** 1 Department of Biological Sciences, Faculty of Science and Technology, Al-Quds University, Jerusalem, Palestine; 2 Biotechnology, Conservation and Valorization of Natural Resources Laboratory, Faculty of Sciences Dhar El Mahraz, Sidi Mohamed Ben Abdellah University, Fez, Morocco; 3 Faculty of Medicine, Department of Medical Microbiology, University of Health Sciences, Istanbul, Turkey; 4 Department of Medical Entomology & Vector Control, School of Public Health, Tehran University of Medical Sciences, Tehran, Iran; 5 Bundeswehr Central Hospital, Koblenz, Germany; 6 Faculty of Pharmacy, Al-Quds University, Jerusalem, Palestine; 7 Department of Entomology, Faculty of Science, Ain Shams University, Abbassia, 11566 Cairo Egypt; 8 Department of Zoology, Faculty of Science, University of Sabratha, Sabratha, Libya; 9 Department of Biological Sciences, Galvin Life Science, Eck Institute for Global Health, University of Notre Dame, Notre Dame, IN 46656 USA

**Keywords:** *Phlebotomus papatasi*, Microsatellites, Population structure analysis, Expressed sequence tags

## Abstract

**Background:**

*Phlebotomus papatasi* is considered the primary vector of *Leishmania major* parasites that cause zoonotic cutaneous leishmaniasis (ZCL) in the Middle East and North Africa. *Phlebotomus papatasi* populations have been studied extensively, revealing the existence of different genetic populations and subpopulations over its large distribution range. Genetic diversity and population structure analysis using transcriptome microsatellite markers is important to uncover the vector distribution dynamics, essential for controlling ZCL in endemic areas.

**Methods:**

In this study, we investigated the level of genetic variation using expressed sequence tag-derived simple sequence repeats (EST-SSRs) among field and colony *P. papatasi* samples collected from 25 different locations in 11 countries. A total of 302 *P. papatasi* sand fly individuals were analyzed, including at least 10 flies from each region.

**Results:**

The analysis revealed a high-level population structure expressed by five distinct populations A through E, with moderate genetic differentiation among all populations. These genetic differences in expressed genes may enable *P. papatasi* to adapt to different environmental conditions along its distribution range and likely affect dispersal.

**Conclusions:**

Elucidating the population structuring of *P. papatasi* is essential to *L. major* containment efforts in endemic countries. Moreover, the level of genetic variation among these populations may improve our understanding of *Leishmania*–sand fly interactions and contribute to the efforts of vaccine development based on *P. papatasi* salivary proteins.

**Graphical Abstract:**

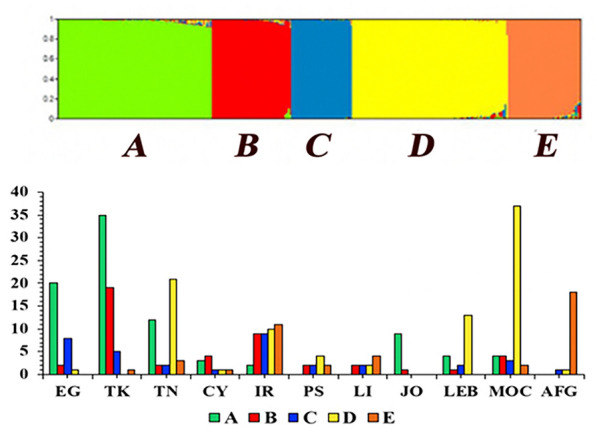

## Background

Leishmaniasis is a disease complex caused by species of *Leishmania* parasites that are transmitted by various sand fly species of the genus *Lutzomyia* in the New World and *Phlebotomus* in the Old World, with *Phlebotomus papatasi* transmitting zoonotic cutaneous leishmaniasis (ZCL). The World Health Organization’s recent fact sheet estimates approximately 0.7–1 million new cases of cutaneous leishmaniasis (CL) annually, with the countries of the Middle East and North Africa—Afghanistan, Algeria, Ethiopia, Iran, Sudan, and Syria—considered to be endemic for CL, in addition to Morocco, Pakistan, Saudi Arabia, Tunisia, and Turkey that are considered to carry a high CL burden [1]. Despite the high burden, leishmaniasis is still treated as a neglected disease [2].

In light of these concerning reports about the global incidence of CL, control programs should be implemented and directed toward the sand fly vectors, taking into account the clear genetic makeup of *P. papatasi* populations in the targeted region; otherwise, the use of insecticides will be of low validity, especially after the emergence of insecticide-resistant sand fly species including *P. papatasi* [3–7].

Population genetic analysis can provide valuable information to characterize genetically distinct populations and gene flow between discrete populations. If gene flow between populations is high, the sand fly vectors may have a high probability of colonizing new sites, increasing the expansion and distribution of the sand fly species and impacting disease transmission. Population structure analysis can also provide vital information about a vector’s biology that may influence its distribution and dispersal capacity, thus increasing the efficiency of vector control programs and implementation of novel vector control strategies. Moreover, identifying patterns of heterogeneity and different population structures of sand fly vectors may reveal the existence of cryptic populations that can alter disease transmission and potentially modify predictions of disease dynamics [8–10]. In addition, inter-populational variation among vectors in terms of vector competence has serious consequences for the susceptibility of *P. papatasi* females to parasites, changes in biting behavior and longevity, and the ability to adapt to changing environmental or ecological conditions [11–13].

As vaccine development for leishmaniasis based on proteins expressed in sand flies is considered a vital strategy, with most efforts based on *P. papatasi* salivary gland proteins [14, 15], assessing the genetic variability of sand fly proteins using efficient transcriptome markers is critical. The development of vaccines focused on genetically homogeneous sand fly populations will be more effective, since variation in the expression of genes can reflect the possibly variable proteins that influence the behavior of the *P. papatasi* sand fly toward the host and at the same time facilitate the infection of the parasite in the host blood [16, 17]. In addition, identifying variable sand fly individuals may extend to the molecules that are likely important factors of sand fly–vertebrate host and sand fly–*Leishmania* interactions [18]. Given their geographical range and limited dispersal ability, it is expected that different *P. papatasi* populations will demonstrate greater genetic diversity, complicating the development of a sand fly saliva-targeted vaccine [19–22].

*Phlebotomus papatasi* sand flies are considered intercontinental, with a huge geographical range, including different climatic and ecological conditions that could be favorable for local adaptation. We previously identified panels of nuclear and transcriptome markers that were validated as useful tools for population analysis of *P. papatasi* sand flies and extensively used to characterize *P. papatasi* populations [21, 23–26]. Using a panel of five microsatellite markers, we identified population structure and substructure from *P. papatasi* flies collected from 15 countries; however, no clear geographical structuring was evident, likely due to both the low number of markers and the low number of *P. papatasi* flies collected in each population [23, 27]. Studies using mitochondrial DNA markers such as cytochrome b and cytochrome c oxidase 1 were also able to determine closely related and coexisting *P. papatasi* populations in the Middle East [21, 24, 28, 29]. Other genetic markers used for this sand fly species proved to be of low efficiency and reliability and could not be used for clear and accurate population structure analysis [30, 31].

Taking advantage of the completion of the genome sequencing project for *P. papatasi* and the availability of the whole-genome sequence data of different *P. papatasi* individuals from three different countries, single nucleotide polymorphisms (SNPs) were identified and population structure analysis was carried out [32]. Although whole-genome sequencing provided a deeper analysis of the genome, the advantage of microsatellites lies in their highly polymorphic nature and easy development, especially based on mining sequences.

Microsatellites are the molecular marker of choice for genetic analysis because of their high power of resolution. Microsatellites evolve much faster than mitochondrial or nuclear genes, making these markers particularly useful for inferring genetically variable populations at a finer geographical and evolutionary scale. In addition, the development of microsatellites from expressed sequence tags (EST) has been shown to be a feasible option for obtaining high-quality genetic markers for various organisms, including *P. papatasi* sand flies and other insect species [33–36].

Here, we used our previously developed and validated panel of 14 EST microsatellite markers [26] to investigate the genetic structure and to identify patterns of genetic variability among colony- and field-collected *P. papatasi* sand flies obtained from different countries in Asia and Africa.

## Methods

### Sand fly samples

**Fig. 1:**
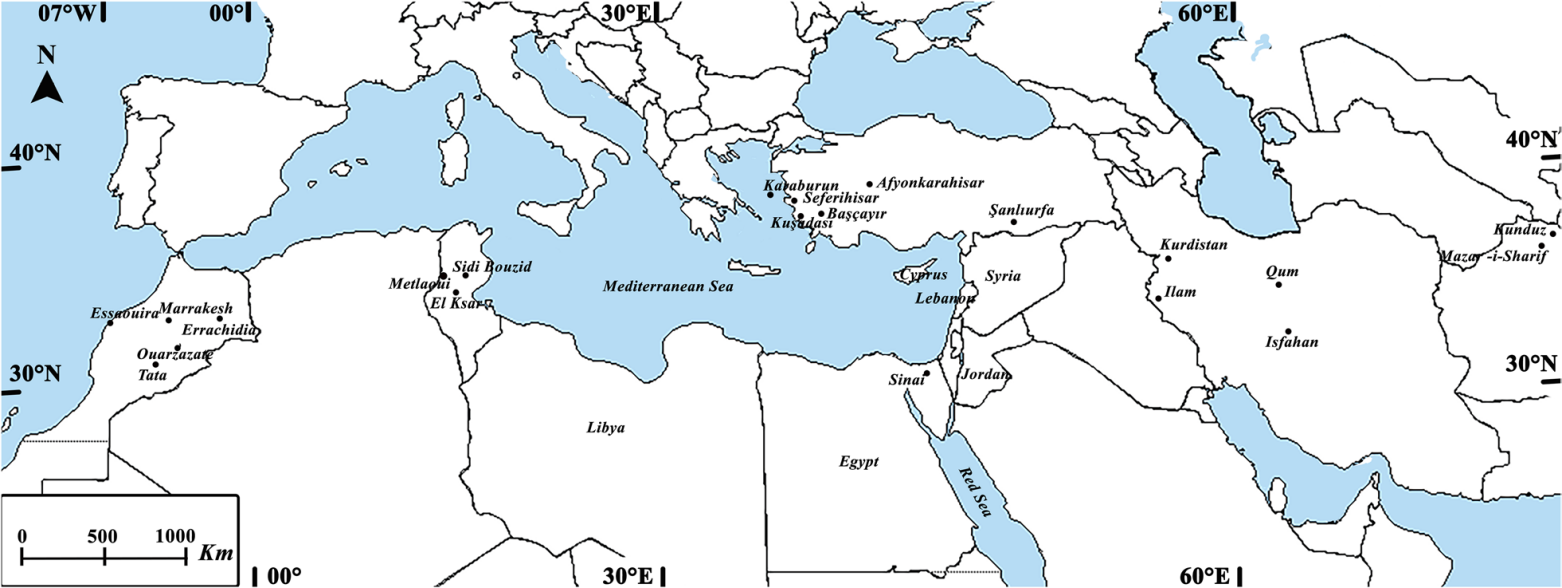
Study locations where *P. papatasi* sand flies were collected and provided for this project

*Phlebotomus papatasi* sand flies were collected from the field or provided by contributors from reared *P. papatasi* colonies from the following countries: Egypt (two colonies and one field collection), Turkey (one colony and six field collections from Seferihisar, Izmir, Kusadasi, Karaburn, Afyonkarahisar, Şanlıurfa, and Başçayır/Aydın), Cyprus (one field collection), Tunisia (three field collections; one from Métlaoui and two from Kasr-Gafsa), Iran (four field collections from Isfahan, Qum, Ilam, and Kurdistan), Libya (one field collection), Jordan (colony), Lebanon (two field collections from Memzaryah and Kafr), Morocco (five field collections from Tata, Erradidia, Quardaza, Essaonira, and Marrakesh), and Palestine (one field collection). A total of 302 *P. papatasi* sand fly individuals were analyzed, including at least 10 flies from each region (Fig. 1). For field-collected sand flies, *P. papatasi* males were identified based on taxonomic characteristics using entomological keys.

### DNA extraction and microsatellite genotyping

DNA was extracted from individual sand flies using a DNA extraction kit (Invitrogen, Carlsbad, CA, USA), following the manufacturer’s instructions. Fourteen EST microsatellite primers were developed and validated previously by our group and used to genotype each individual fly [26].

The polymerase chain reactions (PCRs) were carried out in a 25 μl reaction mixture containing 2.5 μl 10× PCR buffer, 0.5 μl deoxynucleoside triphosphate (dNTP) mixture, 0.15 μl of *TaKaRa Taq*, 1.2 μl of template DNA, and 0.5 μM of each primer. For PCR amplification, DNA was denatured at 94 °C for 5 min followed by 35 cycles (94 °C for 45 s, annealing for 40 s, 72 °C for 45 s), and a final extension at 72 °C for 7 min. Polymorphisms were evaluated by separating PCR products on high-resolution 3.5% MetaPhor agarose gel (Lonza, Rockland, ME, USA). For accurate sizing of the polymorphic PCR products, the forward primers were labeled with 5′-fluorescent dyes (D2–D4). The PCR products were then analyzed using an automated CEQ™ 8000 sequencer (Beckman Coulter, Fullerton, CA, USA), and the fragment sizes were analyzed using its fragment analysis tool. The number of repeats was calculated based on the standards that were sequenced previously, and the number of repeats was calculated based on these standards.

### Population structure analysis

Population structure analysis was carried out using PopCluster software [37], where a Bayesian clustering algorithm was run using an admixture model without any predefined information about the origin of the data for each *K* from 1 to 10, with *K* being the number of populations. Genetically distinct populations were identified based on allele frequencies, and each individual was placed into an appropriate *K* population regardless of its geographical origin. Individuals could be assigned to multiple clusters, with the membership coefficients of all those clusters summing to 1. To assess the most appropriate number of *K* clusters, the second-order rate of change (*DLK2)* value was calculated using the admixture model from *K* = 1 to *K* = 10 for 10 consecutive runs for each *K*. The *DLK2* from the *K* = 1 to 10 curve was obtained directly as an output from the PopCluster program. Genetic differentiation (*F*_ST_), inbreeding coefficient (*F*_IS_), and migration index (*F*_IT_) between populations were also analyzed using the PopCluster program.

## Results

A total of 302 *P. papatasi* sand flies were genotyped using 14 EST genetic markers previously developed and validated by our group [26]. The origins, locations, and distribution of the samples are shown in Fig. 1. On average, 10 sand fly individuals were used from each locality or colony. Only male *P. papatasi* sand flies were analyzed from field populations, and both males and females were analyzed for colony-originated flies.

### Population structure analysis

**Fig. 2:**
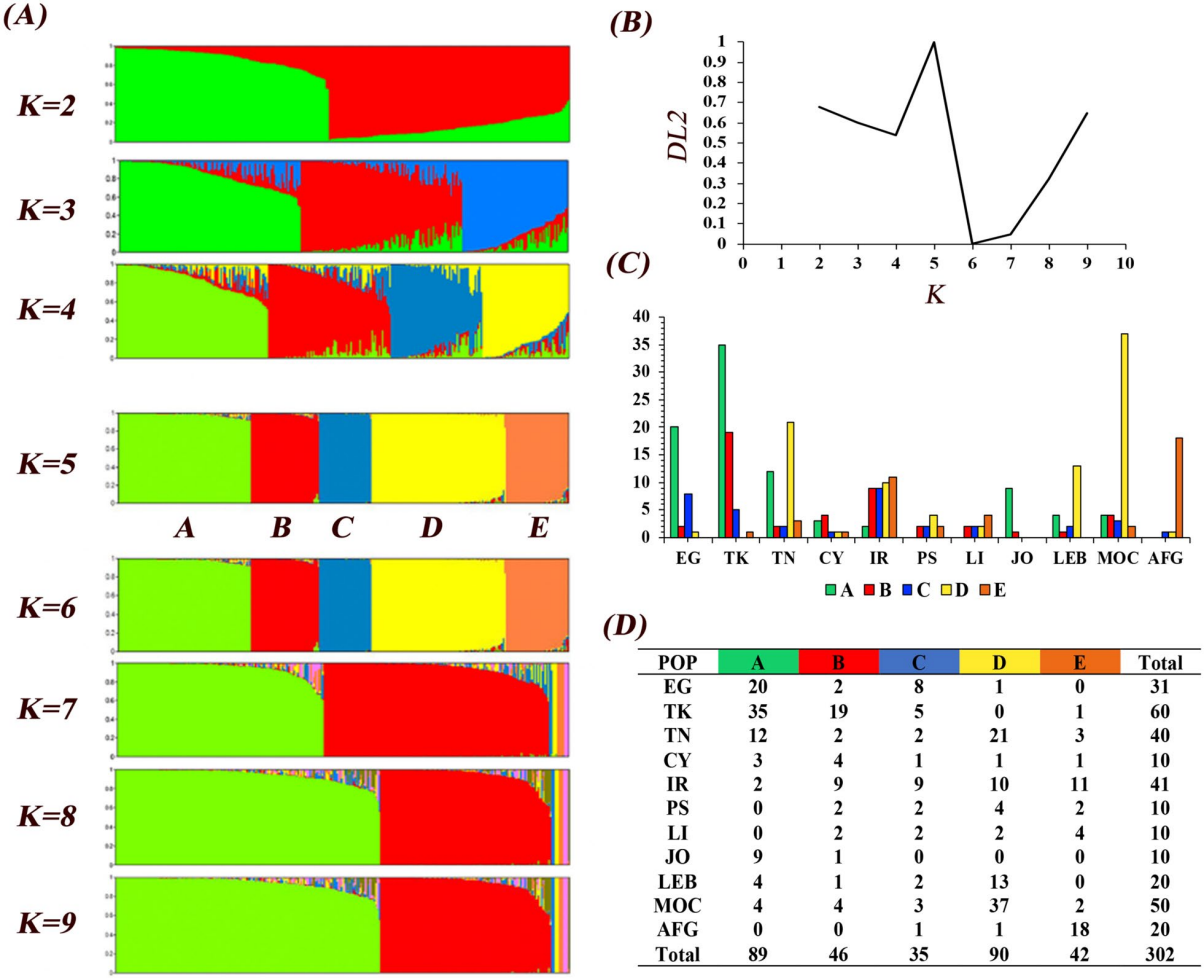
Analysis of the population structure of 302 *P. papatasi* sand fly individuals from 11 countries and genotyped using 14 microsatellites. **A** The output of PopCluster run from *K* = 1 to *K* = 9. **B** The optimal *K* which is represented by a peak of *DLK2* over *K* = 5. **C** The total number of individuals in *y* axes assigned to *P. papatasi* individuals from each country. **D** The total number of individual flies per country assigned to the resolved populations at *K* = 5

To detect the population structure of *P. papatasi*, all data were analyzed in PopCluster software using an admixture model. The whole dataset was grouped into five main populations, A–E, which was confirmed by a peak of *DLK2* that reached a maximum value over *K* = 5 (Fig. 2B). Out of 302 individuals, 89 individuals were grouped into the A population, with flies originating from Turkey (35), Egypt (20), Tunisia (12), Jordan (9), Morocco (4), Lebanon (4), Iran (2), and Cyprus (3) (Fig. 2C). Forty-six individuals were grouped into the B population (Fig. 2D), originating from Turkey (19), Iran (9), Cyprus (4), Morocco (4), Egypt (2), Tunisia (2), Palestine (2), Libya (2), Jordan (1), and Lebanon (1). An interesting finding is the existence of a genetically mixed population (population C) in which individuals from almost all locations were represented, with a total of 35 individuals originating from Egypt (8), Iran (9), Turkey (5), Morocco (3), Afghanistan (1), Cyprus (1), Tunisia (2), Palestine (2), Libya (2), and Lebanon (2). Population D had 90 individuals, originating from Morocco (37), Tunisia (21), Lebanon (13), Iran (10), Palestine (4), Libya (2), Afghanistan (1), Cyprus (1), and Egypt (1). Finally, population E had 42 individuals originating from Afghanistan (18), Iran (11), Libya (4), Tunisia (3), Morocco (2), Palestine (2), Turkey (1), and Cyprus (1).

The genetically mixed population (population A) dominated by individuals from the East Mediterranean (Jordan, Egypt, and Turkey) with equal kinship (genetic relatedness) values and very few individuals from other regions may be considered as individual variation among *P. papatasi* sand flies. Approximately 64.4% of individuals assigned to group D originated from two North African countries, Morocco and Tunisia (41.1% and 23.3%, respectively), while other sand fly individuals from Morocco (13/50, 26%) scattered in other groups. It was interesting to discover that the sand fly individuals from Iran are the most heterogeneous, with individuals from this country represented in all populations (A–E). Remarkably, 18 out of 20 *P. papatasi* individuals from Afghanistan were grouped in a special population E, which also includes 11 individuals from Iran (Fig. 2C).

### Genetic differentiation among subpopulations

Among the five populations, *F*_ST_ values range from 0.0914 (between populations B and A) to 0.1894 (between populations E and A). The *F*_ST_ estimates within populations range from 0.0722 to 0.1848 for populations B and A, respectively (Table 1), indicating that each population is a true population.

**Table 1:** Differentiation estimates: *F*_ST_ values for each inferred population in admixture analysis within population (underlined values in diagonal square matrix) and between each pair of populations A–E

Population	A	B	C	D	E
A	0.1848				
B	0.0914	0.0722			
C	0.1089	0.1149	0.0955		
D	0.1623	0.1344	0.1241	0.1165	
E	0.1894	0.1656	0.1404	0.1767	0.1114

Inferred *F*_ST_ values range from 0.045 for the B population to 0.146 for the A population. On the other hand, population C has the highest *F*_IS_ and *F*_IT_ values (0.636, 0.663, respectively), while population A has the lowest *F*_IT_ and *F*_IS_ values (0.477, 0.552, respectively), (Table 2).

These *F*-statistic results demonstrate that moderate genetic differentiation exists among all populations, with the average *F*_IT_ value over all populations at 0.571, which indicates that there are population differences among the individuals screened

## Discussion

While *P. papatasi* sand flies have been believed to be genetically heterogeneous through their wide distribution range [21, 23, 24, 27, 30, 38, 39], we revealed in this study the existence of complex genetic structuring in populations of this species. Ecological determinants such as topography, climate, soil conditions, and plant cover can influence the distribution of *P. papatasi* sand flies [40]. Moreover, anthropogenic changes and human migration can also lead to an increase in sand fly dispersal and hence disease transmission [41]. Therefore, the existence of the population structure of *P. papatasi* may have a great influence on *L. major* populations circulating in countries (Table 2).

**Table 2:** Estimated *F* statistics for each inferred population. The first column is the population index and the average (Av.); the second column (Total individuals) is the number of individuals belonging to the corresponding population; the other columns represent the inferred *F*_ST_, *F*_IS_, and *F*_IT_ values for each of the inferred populations A–E

Population	Total individuals	*F*_ST_	*F*_IS_	*F*_IT_
A	89	0.146	0.477	0.552
B	46	0.045	0.502	0.524
C	35	0.072	0.636	0.663
D	90	0.079	0.515	0.554
E	42	0.065	0.533	0.564
Av		0.081	0.533	0.571

Using transcriptome microsatellites, this report describes complex genetic structuring among field and colony *P. papatasi* sand flies collected from 11 countries. The genetic variation reveals the existence of a high-level population structure expressed in five distinct populations and proved by a peak of *DLK2* over *K* = 5. This great genetic difference in expressed genes may enable *P. papatasi* to adapt to different environmental conditions along its distribution range. The genetic variation among populations may affect the dispersal and possibly control of this species through the upregulation or variation in insecticide-resistant genes that is common among vectors of infectious diseases [42].

The high genetic diversity among *P. papatasi* populations is not new, as it was previously reported using nuclear and mitochondrial markers [21, 24–27, 43–45]; however, this is the first report of clear and well-separated populations using transcriptome microsatellite markers, which have the benefit of revealing more levels of genetic variation among populations based on expressed genes.

The existence of two main populations (A and B) which are highly dominated by individuals from the East Mediterranean—Egypt, Turkey, and Cyprus—was previously reported [24, 27]. Population C includes intermixed individuals from almost all the analyzed countries, possibly indicating a higher level of genetic variation for some individuals originating from all countries. Most of the Tunisian and Moroccan individuals clustered in population D, which represented the North African region. Although some of the Tunisian individuals clustered in the East Mediterranean populations (A and B), individuals from Tunisia may be considered transition populations between East and West Mediterranean regions. Future studies on more individuals from different areas of Tunisia will help reveal the genetic makeup of *P. papatasi* sand flies in this country. Similarly, 13 out of 20 individuals from Lebanon clustered in the D population, which was also dominated by individuals from Morocco and Tunisia, indicating the existence of more than one population in this small country that has different topographical features, and hence *P. papatasi* populations seem to be distributed with elevation [46, 47]. Remarkably, almost all individuals from Afghanistan (18/20) were clustered in population E, which also included some individuals from neighboring Iran (11 out of 41).

The assignment of individuals to populations carried out by PopCluster software revealed that the Iranian individuals were heterogeneous, as they were assigned almost equally to four populations (B–E). Based on five nuclear microsatellite markers and mitochondrial genes, it was reported previously that two subpopulations in Iran coexisted in the country [27, 45, 47]. Twelve out of 40 individuals from Tunisia were assigned to population A, in addition to the rest (21 out 40) assigned to population D, which also predominated with individuals from Morocco. For East Mediterranean populations (A and B), individuals represented about one third of the total collected individuals and were dominated by East Mediterranean countries, with very few individuals from Morocco, Tunisia, and Libya, which may be considered as individual variation.

More extensive country-wide collections for *P. papatasi* will be carried out in the future for Iran, Tunisia, Lebanon, and Morocco, as the assignment of these individuals to more than one population is very prominent in this study. Interestingly, reports have described the existence of morphological differences between different *P. papatasi* males collected from different areas in Morocco [10]. Further research should be directed toward analysis of more individuals from countries with a small sample size like Libya, Cyprus, Jordan, and Palestine.

### Impacts of vaccine development based on *P. papatasi* salivary proteins

*Phlebotomus papatasi* salivary proteins are good candidates for the development of an effective vaccine, as saliva facilitates *Leishmania* infection to the host by its immunomodulatory activity, and at the same time it can be protective; repeated exposure to sand fly bites is believed to protect individuals from *Leishmania* infection [48–50]. Our work presented here indicates a substantial population structure that suggests multiple subpopulations. While our work did not strictly assess genes that encode known salivary proteins, previous work has identified substantial genetic variation in salivary genes in the Middle East [21, 38]. In our opinion, genetic diversity among populations of *P. papatasi* may be considered an obstacle to the development of a single protein-based vaccine due to the high number of polymorphism or allele-specific protein variations. Continued surveillance of the population dynamics of *P. papatasi* will allow the identification and characterization of more potential targets and advance vaccine progress, especially when concerning the development of a geographically broad-based vaccine and not under positive selection pressure [51]. Proteins conserved across populations with low levels of population variability demonstrate great potential as vaccine candidates [52].

### Impact on *P. papatasi* dispersal

The high genetic diversity revealed by the existence of population structure likely enables *P. papatasi* sand flies to adapt to different environmental conditions and breed with other local or mobile populations and subpopulations to maintain considerable genetic heterogeneity. In recent reports, high numbers of *P. papatasi* sand flies were captured in agricultural areas and very few in residential areas, even though the existence of a few sand flies in the proximity of residential areas is sufficient to cause biting nuisance and transmission of *Leishmania* infection [53].

In studies using cytochrome oxidase and other genetic markers, *P. papatasi* populations from Morocco have been reported to be genetically heterogeneous [39, 43]. In addition, variations among *P. papatasi* populations from Morocco have been documented using morphometric studies [54]. The population structure analysis conducted here revealed the existence of genetically mixed populations, with one population from Morocco (Errachidia) grouped in the B population. This result provides additional evidence of a probable demographic expansion of *P. papatasi* and the existence of a complex scenario of *P. papatasi* populations in Morocco, which has been previously reported [21, 24, 27]. *Phlebotomus papatasi* sand flies may have experienced a period of isolation due to geographical barriers like the high Atlas Mountains. The high-elevation mountains usually generate heterogeneous habitats, where reproductive isolation in spatially discrete populations likely occurs [43, 54, 55]. It is worth mentioning that the numbers of individuals from each studied locality are comparable (approximately 10 individuals); however, the total number of individuals assessed differs among countries due to the availability of sand flies.

The simple sequence repeats (SSRs) developed from ESTs may play an adaptive and evolutionary role through induced changes in amino acid sequence, protein length, three-dimensional (3D) structure, or regulation of gene expression. The transcriptome microsatellites encompass many important genes, including transcription factors, epigenetic modulators, cell cycle controllers, and signaling which regulate growth and development. The high and positive *F*_IS_ values in all *P. papatasi* populations indicated that these populations have different degrees of inbreeding.

## Conclusions

*Phlebotomus papatasi* sand flies analyzed in this study displayed abundant genetic diversity and genetic differentiation. The high level of genetic diversity suggests that this species has a strong capability to adapt to the changes in environmental conditions in its distribution range. Understanding *P. papatasi* genetic variation aids in understanding complex interactions that impact vectorial capacity.

The existence of genetic populations should be considered for planning future control measures and for understanding leishmaniasis outbreaks and transmission dynamics of *Leishmania* parasites among human populations. The genetic complexity of *P. papatasi* sand flies can affect the flow of genes, vector competence, and insecticide resistance. However, other factors can affect sand fly population dynamics and genetic structure patterns, such as microclimate, agricultural activity, increasing urbanization, and global warming. Further population structure analysis of *Leishmania* vectors should be conducted, monitoring changes over time, especially when insecticides are introduced and in light of climate and land use changes. Additional research should be conducted in regions of higher *P. papatasi* heterogeneity, trying to link the existence of different populations with disease epidemiology and severity. The search for a vaccine based on *P. papatasi* salivary proteins must consider this high heterogeneity, as antigenic diversity of salivary proteins and extensive population structure may guide the development of an effective vaccine.

## Data Availability

No datasets were generated or analyzed during the current study.
